# From Childhood Experience to Professional Response: Sequential Mediation of Well-Being and Cognitive Flexibility Among South Korean Early Childhood Educators

**DOI:** 10.3390/bs16050726

**Published:** 2026-05-08

**Authors:** Youme Lee, Misuk Cha, Young-Eun Lee

**Affiliations:** 1 Department of Education, Graduate School of Education, Gachon University, Seongnam-si 13120, Republic of Korea; 2 Department of Social Welfare, Graduate School of Public Policy and Civic Engagement, Kyung Hee University, Seoul 02447, Republic of Korea; 3 Department of Early Childhood Education, College of Social Science, Gachon University, Seongnam-si 13120, Republic of Korea

**Keywords:** maternal unsupportive responses, early childhood educators, unsupportive responses to young children, subjective well-being, cognitive flexibility, intergenerational transmission

## Abstract

This study investigated the influence of early childhood educators’ childhood experiences of maternal unsupportive responses on their current reactions to children’s negative emotional expressions, focusing on the sequential mediating roles of subjective well-being and cognitive flexibility. A sample of 302 early childhood educators in South Korea completed an online survey. Data were analyzed using SPSS 26.0 and the PROCESS macro (Version 4.2, Model 6). The findings indicated that maternal unsupportive responses in childhood significantly and positively predicted educators’ current unsupportive responses to children (β = 0.37, 95% CI [0.21, 0.37]). While the simple mediating effect of subjective well-being was not significant (β = −0.01, 95% CI [−0.06, 0.03]), cognitive flexibility significantly mediated this relationship (β = 0.04, 95% CI [0.00, 0.07]). Furthermore, a significant sequential mediating effect was identified (β = 0.04, 95% CI [0.02, 0.07]): educators who reported more maternal unsupportive responses in childhood showed lower subjective well-being and, in turn, lower cognitive flexibility, which was associated with more frequent unsupportive reactions toward children’s negative emotions. These results are consistent with an intergenerational pattern of emotional socialization, suggesting the potential role of an affective–cognitive mechanism. The study suggests that teacher education and support policies should integrate reflective practices on childhood experiences with programs that enhance emotional well-being and cognitive flexibility, thereby improving the quality of teacher–child interactions.

## 1. Introduction

Early childhood is a critical period for laying the foundation of social-emotional development, during which young children develop emotional regulation skills through emotional interactions with caregivers (Denham et al., 2003; Morris et al., 2007). Traditionally, these interactions centered primarily on relationships with parents within the home. However, with the increase in dual-income households and the strengthening of society’s responsibility for childcare, young children now spend longer hours in educational institutions from an earlier age (Katsarou et al., 2026; Spilt & Koomen, 2022). Consequently, the role and responsibility of early childhood educators as agents of emotional socialization supporting young children’s emotional stability and development have become increasingly important (Denham et al., 2012; Kwon & Kim, 2025).

Within the emotional socialization framework (Eisenberg et al., 1998), caregivers’ responses to children’s emotional expressions play a key role in the development of emotional regulation skills and social competence. These principles equally apply to the teacher-child relationship (Denham et al., 2017; Fatahi et al., 2022; Poulou & Denham, 2023). Young children frequently express not only positive emotions but also negative emotions such as anger, frustration, and crying. These expressions of negative emotion are not merely problematic behaviors to be suppressed but meaningful signals of internal states that warrant sensitive responses (Cekaite & Ekström, 2019). Therefore, when educators respond sensitively and demonstrate supportive attitudes when children express negative emotions, it can promote the development of children’s emotional regulation abilities and social competencies (Bassett et al., 2017; Denham et al., 2022; Pakarinen et al., 2020; Wilhelmsen et al., 2025).

However, research consistently indicates that early childhood educators are receptive to children’s positive emotional expressions but find it challenging to guide them in expressing negative emotions (Cekaite & Ekström, 2019; Denham et al., 2012; Zinsser et al., 2014). They sometimes exhibit responses that ignore the severity of emotional distress or resort to punishment (Byun & Jeon, 2023; Denham & Bassett, 2019). Such unsupportive responses can negatively impact young children’s emotional development (Fabes et al., 2002; Jeon et al., 2016; Katsarou et al., 2026; Li et al., 2022), necessitating an examination of contributing factors that extends beyond immediate classroom conditions to educators’ own developmental histories.

## 2. Literature Review and Theoretical Hypotheses

Among such developmental factors, childhood experiences with caregivers merit particular attention because emotional interaction patterns can be transmitted across generations (Eisenberg et al., 1998; Morris et al., 2007). Studies indicate that educators’ early experiences with their own caregivers affect the quality of teacher-child interactions (Jang & Hong, 2024; Rancher & Moreland, 2023). However, two important questions remain unanswered. First, although the intergenerational transmission of emotional socialization is well-documented in parent–child research (Girod et al., 2025; Leerkes et al., 2020), this process has received limited attention in the teacher-child context, and the few available studies have addressed general teacher-child interaction quality rather than the specific unsupportive responses that constitute emotional socialization. Second, although prior work suggests that the transmission operates through indirect psychological processes (Leerkes et al., 2020), the specific intrapersonal resources through which this occurs in educators have yet to be systematically examined.

According to the emotional socialization model (Eisenberg et al., 1998), caregivers’ responses to children’s negative emotions shape how children come to understand, express, and regulate their own emotions. When mothers repeatedly minimize, punish, or display distress in response to their children’s negative emotions, children are likely to develop stable beliefs that such emotions are unacceptable or should be suppressed (Eisenberg et al., 1998; Leerkes et al., 2020). These internalized beliefs and response patterns, acquired through repeated socialization experiences, can persist into adulthood and influence how individuals respond to others’ emotional expressions in subsequent caregiving roles (Leerkes et al., 2020; Milan et al., 2021). Indeed, research on the intergenerational transmission of emotional socialization demonstrates that mothers who recalled unsupportive responses from their own caregivers in childhood were more likely to exhibit unsupportive responses to their own children’s distress, and that this transmission operated primarily through indirect psychological processes rather than direct behavioral replication (Girod et al., 2025; Leerkes et al., 2020).

Building on this evidence, the underlying psychological mechanisms of emotional socialization are unlikely to remain confined to the family of origin. Morris et al. (2007) proposed that parents’ own familial histories and meta-emotion beliefs are internalized and carried into adulthood, where they continue to shape how emotions are socialized in subsequent caregiving relationships. Consistent evidence from interpersonal acceptance-rejection theory further indicates that adults’ remembrances of parental rejection in childhood predict their psychological adjustment and the quality of their subsequent close relationships (Rohner et al., 2020), suggesting that internalized early experiences extend beyond the family of origin into other significant relationships. Critically, this view is not an extension imposed upon Eisenberg et al.’s (1998) foundational framework, but is anticipated within it. Eisenberg et al. (1998) explicitly situated emotional socialization within the activities of “parents and other socializers,” thereby recognizing from the outset that the mechanisms underlying emotional socialization are not exclusive to the parent–child dyad. This inclusive conceptualization implies that the psychological processes through which emotional socialization operates, including modeling, contingent responding, and the communication of emotion-related beliefs, are sufficiently general to be carried and re-enacted across different caregiving roles and relational contexts throughout adulthood.

At the same time, parental and teacher emotional socialization are enacted under contextually distinct conditions and differ along at least three dimensions. First, in the structure of interaction, parental emotional socialization typically unfolds within dyadic parent–child exchanges, whereas teacher emotional socialization occurs within group settings where educators must manage multiple children’s emotions simultaneously and orchestrate peer-mediated emotional dynamics (Wilhelmsen et al., 2025). Second, in professional grounding, parental practices are shaped largely by personal developmental histories and family emotional culture, whereas teacher practices are additionally informed by formal preparation in child development and classroom emotion management (Denham et al., 2017; Jennings & Greenberg, 2009). Third, in developmental goals and temporal scope, parental emotional socialization is embedded in lifelong attachment relationships, whereas teacher emotional socialization operates within bounded programmatic contexts oriented toward children’s social-emotional competence and school readiness (Denham et al., 2022; Pakarinen et al., 2020). These contextual specificities do not preclude the operation of enduring psychological mechanisms carried over from early life; rather, they suggest that such mechanisms may be expressed differently across settings. Formal training and professional norms may shape the surface form of educators’ responses, while internalized early experiences continue to shape the underlying affective and cognitive dispositions that influence how educators respond to children’s emotions in practice.

Considering that mothers have traditionally been the primary agents of emotional interaction in Korean society (Song & Trommsdorff, 2016; Ziehm et al., 2013), the unsupportive responses experienced from mothers likely significantly influenced the formation of educators’ emotional response systems. The present study addresses these gaps by examining early childhood educators as professional caregivers, employing the CCNES framework to assess unsupportive responses across both generations, and investigating trait-level subjective well-being and cognitive flexibility as mediating pathways. We focus on these two resources because they correspond to the affective and cognitive components that are widely recognized as central to emotional socialization. Subjective well-being, as an affective resource, shapes how adults process and respond to emotional signals (Fredrickson, 2001), whereas cognitive flexibility, as a cognitive resource, supports the reappraisal and adaptive interpretation of emotionally demanding situations (Diamond, 2013; Dennis & Vander Wal, 2010). Together, these two resources provide a parsimonious account of how early emotional socialization experiences shape adult caregiving behavior. The following sections develop the theoretical rationale for these mediating mechanisms.

First, subjective well-being may mediate the influence of a mother’s unsupportive responses experienced in childhood on an educator’s unsupportive responses. Subjective well-being refers to an individual’s overall satisfaction with their life and positive emotional experiences (Diener, 1984). Within the emotional socialization framework, repeated exposure to unsupportive parental responses during childhood can undermine the development of healthy emotion understanding and a positive affective stance toward one’s own emotional experiences (Eisenberg et al., 1998). Such experiences have been associated with lower psychological well-being and maladjustment in adulthood (Klein et al., 2020; Peter et al., 2025). Separately, research has demonstrated that early childhood educators with compromised well-being exhibit lower-quality emotional interactions with children (Markowitz et al., 2024) and more negative responsiveness toward children’s emotional expressions (Byun et al., 2022). Therefore, maternal unsupportive responses experienced by early childhood educators during childhood may negatively impact their subjective well-being, potentially leading to unsupportive reactions to children’s expressions of negative emotions.

Second, the effect of a mother’s unsupportive responses experienced during childhood on an educator’s unsupportive responses may be mediated by cognitive flexibility. Cognitive flexibility refers to the ability to flexibly adjust thoughts and behaviors to meet the demands of changing situations, and it is a core subcomponent of executive function (Diamond, 2013; Hohl & Dolcos, 2024). Repeated exposure to unsupportive responses may limit opportunities to practice reappraising emotionally challenging situations from multiple perspectives—a core component of cognitive flexibility (Dennis & Vander Wal, 2010). Such constrained developmental experiences may, in turn, shape professional responses; among Korean early childhood educators, cognitive flexibility was positively associated with the quality of teacher–child interactions (H. S. Park & Suh, 2023). These findings suggest that unsupportive responses experienced from one’s mother during childhood may impair educators’ cognitive flexibility, potentially influencing their unsupportive reactions to children’s expressions of negative emotions.

These two psychological resources, however, do not operate in isolation. According to Fredrickson’s (2001) Broaden-and-Build Theory, positive emotional states broaden an individual’s momentary thought-action repertoires—expanding the range of thoughts, actions, and attentional focus available in a given moment—and over time, these broadened repertoires build enduring personal resources, including cognitive flexibility and creative problem-solving capacities. Conversely, diminished positive affect narrows the thought-action repertoire, potentially constraining the cognitive resources available for adaptive responses. Supporting this theoretical linkage from a neurocognitive perspective, Diamond (2013) reported that emotional stress and negative emotional states impair prefrontal cortex function, hindering the efficient exercise of executive functions, including cognitive flexibility. She emphasized that emotional well-being is a core condition for cognitive functioning.

These theoretical and empirical grounds suggest the possibility of a sequential mediating pathway: maternal unsupportive responses experienced in childhood affect cognitive flexibility via subjective well-being, ultimately leading to unsupportive responses to the child’s negative emotional expressions. In other words, the educator’s psychological resources act sequentially to shape the educator’s professional responses. However, studies that comprehensively examine educators’ unsupportive responses within a developmental context are difficult to find. Therefore, this study aims to clarify the process by which childhood nurturing experiences influence educators’ unsupportive responses through subjective well-being and cognitive flexibility as sequential mediators, while controlling for age, education level, and teaching experience, which have been identified as potential correlates of teachers’ emotional responses (Byun & Jeon, 2023; Byun et al., 2022; Kim et al., 2019).

Based on the theoretical framework and prior research discussed above, the following hypotheses were established:

**H1.**  *Maternal unsupportive responses experienced during childhood will significantly influence early childhood educators’ unsupportive responses to children’s negative emotional expressions.*

**H2.**  *Subjective well-being will mediate the relationship between maternal unsupportive responses in childhood and educators’ unsupportive responses.*

**H3.**  *Cognitive flexibility will mediate the relationship between maternal unsupportive responses in childhood and educators’ unsupportive responses.*

**H4.**  *Subjective well-being and cognitive flexibility will sequentially mediate the influence of maternal unsupportive responses in childhood on educators’ unsupportive responses.*

**Figure 1:**
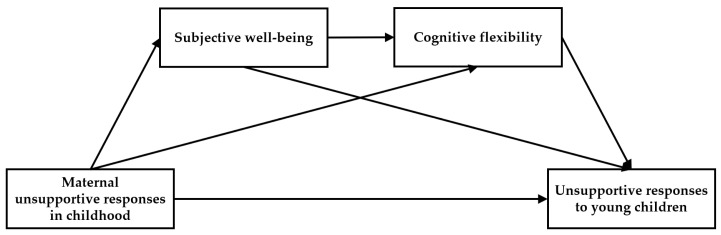
Sequential mediation research model.

The sequential mediation model used in this study is presented in Figure 1.

## 3. Materials and Methods

### 3.1. Research Procedure and Participants

Prior to the main survey, a preliminary study was conducted with 10 early childhood educators to review the suitability of the questionnaire’s structure and the clarity of the item content. Following this, the main survey was administered to a sample of 305 early childhood educators working in kindergartens and childcare centers across the Seoul, Gyeonggi, and Gyeongsang regions of South Korea. Participants were recruited through convenience and snowball sampling with the cooperation of the heads of participating kindergartens and childcare centers. Eligibility was limited to lead teachers of classes of 3- to 5-year-olds; teachers in administrative-only positions and those on leave were not eligible. Data collection was performed via an online platform. Of the initial responses, three were excluded due to careless responding, yielding a final analytic sample of 302 questionnaires.

The demographic characteristics of the participants are summarized in Table 1.

### 3.2. Measures

#### 3.2.1. Maternal Unsupportive Responses in Childhood

Maternal unsupportive responses experienced in childhood were assessed using the Coping with Children’s Negative Emotion Scale (CCNES) developed by Fabes et al. (1990). This study utilized the Korean version adapted by S. Y. Park et al. (2011), with item wording modified from a parent-report format to a retrospective self-report format assessing educators’ childhood experiences with their mothers. The measure focuses on the unsupportive response sub-dimension, which comprises 36 items across three sub-factors: minimization, punitive, and distress reactions. Specifically, minimization reactions involve treating the child’s negative emotions as insignificant or diminishing their value; punitive reactions are characterized by responding to emotional expressions with punishment; and distress reactions involve the mother exhibiting her own stress or anxiety in response to the child’s negative feelings. The original 7-point response format was replaced with a 5-point Likert scale, ranging from 1 (not at all) to 5 (very much so), to enhance response quality and reduce participant fatigue among Korean early childhood educators. Prior research indicates that scales with different numbers of response categories yield comparable internal structure, including Cronbach’s alpha and factor loadings (Leung, 2011). The scores for each sub-factor were averaged, with higher scores indicating a higher frequency of maternal unsupportive responses experienced during childhood. Although retrospective self-reports are subject to recall bias and mood-congruent memory effects (Baldwin et al., 2019), they remain strongly predictive of adult psychological outcomes (Baldwin et al., 2024).

To evaluate the factor structure of the adapted measure, three balanced parcels per sub-factor were constructed following the item-to-construct balance procedure (Little et al., 2002). Parceling was adopted to improve estimation stability, given the large number of items, and to maintain a reasonable indicator-to-sample-size ratio. We acknowledge, however, that this approach may obscure item-level misfit and that the goodness-of-fit of individual items cannot be directly evaluated within the parcel-level CFA model. Bearing this limitation in mind, the three-factor CFA with parcel indicators demonstrated excellent fit to the data, χ^2^(24) = 66.18, *p* < 0.001, CFI = 0.98, TLI = 0.96, RMSEA = 0.076 (90% CI [0.06, 0.10]), SRMR = 0.050. All standardized factor loadings were statistically significant, ranging from 0.62 to 0.91 (all *p* < 0.001). In the present study, the Cronbach’s α for this scale was 0.89.

#### 3.2.2. Subjective Well-Being

Subjective well-being was assessed using the Revised Subjective Happiness Scale (SHS) developed by Lyubomirsky and Lepper (1999), specifically the Korean version translated and validated by the Panel Study on Korean Children research team (Korea Institute of Child Care and Education, 2014). This instrument consists of four items designed to evaluate the general level of happiness an individual perceives in their life. For instance, participants are asked to characterize the extent to which they generally consider themselves to be a very happy person. Early childhood educators rated each item on a 7-point Likert scale, ranging from 1 (not at all) to 7 (very much so). The scores for the items were averaged, with higher average values indicating a greater level of subjective well-being. Confirmatory factor analysis was consistent with a unidimensional structure, χ^2^(2) = 5.13, *p* = 0.077, CFI = 0.99, TLI = 0.99, RMSEA = 0.072 (90% CI [0.00, 0.15]), SRMR = 0.021. All standardized factor loadings were statistically significant, ranging from 0.68 to 0.94 (all *p* < 0.001). In the present study, the Cronbach’s α coefficient for this scale was 0.86.

#### 3.2.3. Cognitive Flexibility

Cognitive flexibility was measured using the Cognitive Flexibility Inventory (CFI) developed by Dennis and Vander Wal (2010), which was validated for the Korean context through factor analysis by Heo (2011). This scale consists of 19 items designed to assess the ability to generate diverse alternative explanations for events, devise multiple solutions, and the tendency to perceive challenging situations as controllable. For instance, participants are asked to evaluate the extent to which they consider various options before responding to a difficult situation. Early childhood educators rated each item on a 5-point Likert scale, ranging from 1 (not at all) to 5 (very much so). The scores for the items were averaged, with higher average values indicating a greater level of cognitive flexibility. Confirmatory factor analysis of the two-factor structure yielded the following indices: χ^2^(151) = 431.81, *p* < 0.001, CFI = 0.87, TLI = 0.85, RMSEA = 0.078 (90% CI [0.07, 0.09]), SRMR = 0.081. All standardized factor loadings were statistically significant, ranging from 0.46 to 0.77 (all *p* < 0.001). In the present study, the Cronbach’s α coefficient for this scale was 0.88.

#### 3.2.4. Educators’ Unsupportive Responses to Children’s Negative Emotions

Early childhood educators’ unsupportive responses to children’s negative emotional expressions were measured using the Coping with Children’s Negative Emotion Scale–Teacher Version (CCNES-T; Denham et al., 2017), which was adapted for the Korean context by Son (2022). While the original CCNES-T assesses both supportive and unsupportive responses, this study specifically utilized the unsupportive response sub-dimension, comprising 30 items across three types of reactions: punitive, minimization, and distress. As with the CCNES, the original 7-point response format was replaced with a 5-point Likert scale, ranging from 1 (not at all) to 5 (very much so), to enhance response quality and reduce participant fatigue among Korean early childhood educators. The scores were averaged, with higher average values indicating that the educators exhibit more frequent unsupportive responses to children’s negative emotional expressions.

As with the CCNES, three balanced parcels per sub-factor were constructed following the item-to-construct balance procedure (Little et al., 2002), with the same trade-offs regarding item-level misfit noted above applying to this scale as well. Bearing this limitation in mind, A three-factor CFA with parcel indicators demonstrated that the 5-point modification preserved the original factor structure, χ^2^(24) = 67.39, *p* < 0.001, CFI = 0.97, TLI = 0.96, RMSEA = 0.077 (90% CI [0.06, 0.10]), SRMR = 0.033. All standardized factor loadings were statistically significant, ranging from 0.60 to 0.83 (all *p* < 0.001). In the present study, the Cronbach’s α coefficient for this scale was 0.89.

### 3.3. Analytical Approach

Statistical analyses were performed using R (Version 4.5.3) with the lavaan package (Version 0.6-21; Rosseel, 2012) and IBM SPSS Statistics (Version 26.0) with the PROCESS macro (Version 4.2; Hayes, 2022).

Prior to the primary analyses, confirmatory factor analyses were conducted for all adapted instruments with robust maximum likelihood estimation. Model fit was evaluated using multiple indices (CFI, TLI, RMSEA, SRMR), with convergence across indicators weighted more heavily than any single cutoff (Hu & Bentler, 1999; Marsh et al., 2004). Harman’s single-factor test was also performed to assess potential common method bias (Podsakoff et al., 2003), given that all data were collected through self-report measures.

The primary analyses were conducted in SPSS as follows: First, frequencies and percentages were calculated to identify the general demographic characteristics of the participants. Second, Cronbach’s α coefficients were computed to evaluate the internal consistency and reliability of the measurement instruments. Third, descriptive statistics, including means and standard deviations, were determined for the key variables, and skewness and kurtosis were examined to assess the normality of the data distribution. Fourth, Pearson’s correlation analysis was conducted to examine the bivariate relationships among the study variables. Fifth, multiple regression analysis was performed to examine the effect of maternal unsupportive responses in childhood on educators’ unsupportive responses, controlling for age, education level, and teaching experience; tolerance and variance inflation factor (VIF) values were examined to assess multicollinearity. Finally, the PROCESS macro (Model 6) was used to examine sequential mediation through subjective well-being and cognitive flexibility, with the same demographic covariates, and the significance of the indirect effects was tested using bootstrapping with 5000 resamples. Indirect effects were considered statistically significant if the 95% bias-corrected confidence intervals (CI) did not include zero.

## 4. Results

### 4.1. Descriptive Statistics and Correlation Analysis

Table 2 presents the descriptive statistics and the results of the correlation analysis for the primary and control variables. Following Kline’s (2016) criteria, the assumption of normality was satisfied, as all variables exhibited skewness below an absolute value of 3 and kurtosis below an absolute value of 10. Additionally, the correlation coefficients (*r*) among the independent variables were all below the threshold of 0.80, indicating that no multicollinearity issues were suspected according to Field’s (2013) criteria.

Regarding the correlations among the main study variables, maternal unsupportive responses in childhood were significantly and negatively associated with subjective well-being (*r* = −0.34, *p* < 0.001) and cognitive flexibility (*r* = −0.27, *p* < 0.001), while exhibiting a significant positive correlation with educators’ own unsupportive responses to children’s negative emotions (*r* = 0.39, *p* < 0.001). Furthermore, both subjective well-being (*r* = −0.21, *p* < 0.001) and cognitive flexibility (*r* = −0.37, *p* < 0.001) were significantly and negatively associated with unsupportive responses toward children. Subjective well-being and cognitive flexibility showed a significant positive correlation (*r* = 0.47, *p* < 0.001), consistent with the hypothesized association between these two resources.

### 4.2. Direct Effect of Maternal Unsupportive Responses on Educators’ Current Responses

A simultaneous multiple regression analysis was performed to examine the impact of maternal unsupportive responses experienced in childhood on early childhood educators’ current unsupportive responses to children’s negative emotional expressions, after controlling for age, education level, and teaching experience (Table 3). The regression model was found to be statistically significant (*F* = 15.47, *p* < 0.001) and explained 17% of the variance in the dependent variable (*R*^2^ = 0.17). The Durbin–Watson statistics were 1.96, confirming the independence of the residuals. All tolerance values exceeded 0.58 and all VIF values were below 1.72 (see Table 3), well within the acceptable thresholds of tolerance > 0.10 and VIF < 10 recommended by Field (2013), indicating no multicollinearity concerns.

After accounting for the control variables, maternal unsupportive responses experienced during childhood significantly and positively predicted educators’ current unsupportive responses to children (β = 0.37, *p* < 0.001). This finding suggests that educators who experienced higher levels of unsupportive parenting in their childhood are more likely to exhibit unsupportive reactions when faced with children’s negative emotional expressions in their professional practice.

### 4.3. Sequential Mediating Effects of Subjective Well-Being and Cognitive Flexibility

**Figure 2:**
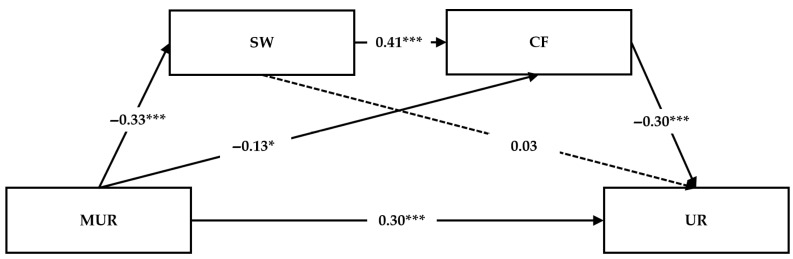
Standardized estimates of the sequential mediating model. *Note*. *N* = 302. * *p* < 0.05; *** *p* < 0.001. MUR, maternal unsupportive responses in childhood; SW, subjective well-being; CF, cognitive flexibility; UR, unsupportive responses to young children. Paths for covariates (age, education level, and teaching experience) are omitted.

Sequential mediation was tested using PROCESS (Model 6) with age, education level, and teaching experience as covariates. The results are summarized in Table 4 and Figure 2.

First, maternal unsupportive responses in childhood significantly and negatively predicted educators’ subjective well-being (β = −0.33, *p* < 0.001; *F* = 15.39, *p* < 0.001). Next, both maternal unsupportive responses and subjective well-being significantly predicted cognitive flexibility (*F* = 18.06, *p* < 0.001). Specifically, maternal unsupportive responses negatively predicted cognitive flexibility (β = −0.13, *p* < 0.05), whereas subjective well-being positively predicted it (β = 0.41, *p* < 0.001).

Finally, the model examining educators’ current unsupportive responses to children’s negative emotions was statistically significant (*F* = 16.10, *p* < 0.001). Maternal unsupportive responses in childhood significantly and positively predicted current unsupportive responses (β = 0.30, *p* < 0.001), while cognitive flexibility was a significant negative predictor (β = −0.30, *p* < 0.001). However, the direct effect of subjective well-being did not significantly predict educators’ current unsupportive responses.

To further validate the hypothesized model, the significance of the indirect effects and the sequential mediation paths—incorporating subjective well-being and cognitive flexibility—was assessed using bootstrapping with 5000 resamples. An indirect effect was considered significant if the 95% bias-corrected confidence interval did not include zero. Results are summarized in Table 5.

The simple mediating effect through subjective well-being was not significant (β = −0.01, 95% CI [−0.06, 0.03]), whereas the simple mediating effect through cognitive flexibility was significant (β = 0.04, 95% CI [0.00, 0.07]). The sequential mediating effect through subjective well-being and cognitive flexibility was also significant (β = 0.04, 95% CI [0.02, 0.07]).

## 5. Discussion

The present study aimed to elucidate the psychological mechanisms underlying the relationship between early childhood educators’ childhood experiences of maternal unsupportive responses and their current unsupportive reactions to children’s negative emotional expressions. The findings are consistent with an intergenerational pattern of emotional socialization and suggest a sequential mediating role of subjective well-being and cognitive flexibility. First, the results indicated that maternal unsupportive responses experienced during childhood significantly and positively predict educators’ current unsupportive responses to children’s negative emotions.

This finding extends the intergenerational transmission of emotional socialization, previously documented in parent–child dyads (Girod et al., 2025; Leerkes et al., 2020), to the professional caregiving context of early childhood education. Critically, whereas prior research identified this transmission within biological parent–infant dyads using situation-specific measures of cognitive and emotional processing (Leerkes et al., 2020), the present findings suggest that similar patterns may emerge in professional caregiving relationships, indicating that such associations may not be confined to biological parent–child dyads.

Notably, this transmission was detected using the same measurement framework—the CCNES—across both generations, enabling a direct comparison of specific response patterns rather than general interaction quality. This approach addresses a limitation identified in prior research, where inconsistent measurement frameworks across generations have made intergenerational comparisons difficult. Furthermore, the fact that this pattern emerged among early childhood educators whose responses are generally expected to reflect formal training rather than personal developmental histories highlights the potential enduring role of early emotional socialization experiences. Early childhood educators’ unsupportive responses, then, may not be reducible to insufficient professional skills alone but may also reflect emotional socialization patterns shaped by their own developmental histories.

Second, the simple mediating effect of subjective well-being was statistically non-significant. Although childhood unsupportive experiences lowered educators’ current well-being, this emotional state did not directly translate into unsupportive classroom responses. This finding reflects the conceptual distance between a global affective measure (SHS) and situation-specific behavioral outcomes (CCNES), suggesting that well-being may require intervening cognitive mechanisms to influence professional responses.

This pattern may be particularly characteristic of Korean early childhood educators due to cultural and professional expectations. Korean cultural context has been described as highly collectivistic, with cultural values emphasizing restraint and perceiving emotions as interpersonal features (Hofstede et al., 2010; Byun & Jeon, 2023). Under such cultural norms, educators may regulate their professional behaviors independently of their personal emotional states. Although conducted with center directors rather than classroom teachers, qualitative research on Korean early childhood education leaders found that educators maintained outward composure while experiencing internal difficulties (Choi & Lee, 2025a). Given that directors and teachers operate within the same institutional and cultural context, similar professional norms may shape classroom teachers’ capacity to differentiate personal well-being from professional conduct. Importantly, this non-significant direct pathway does not imply that well-being is irrelevant to classroom responses. Its role becomes evident in the sequential mediation pathway.

Third, cognitive flexibility significantly mediated the relationship between childhood experiences and current professional responses. Maternal unsupportive responses in childhood were associated with lower cognitive flexibility, which in turn was linked to more unsupportive responses to children. This result aligns with Spilt and Koomen’s (2022) call for research on cognitive mechanisms in teacher-child relationships. In their three-decade review, they highlighted the limited understanding of cognitive processes underlying teachers’ caregiving behaviors. The present findings suggest that cognitive flexibility may serve as one such mechanism. This capacity appears particularly relevant in early childhood education, where educators must respond to children’s emotional expressions within group contexts that demand simultaneous attention to multiple children. When a child expresses anger, for example, interpreting the behavior as an expression of unmet needs rather than defiance requires the educator to inhibit an initial, automatic interpretation and consider an alternative perspective.

The capacity to engage in such reinterpretation, however, may be shaped by one’s own developmental history. When caregivers consistently dismiss or minimize emotional expressions, children may learn that negative emotions are unacceptable and develop a restricted interpretive repertoire rather than flexible appraisal strategies (Cassidy, 1994; Eisenberg et al., 1998). Such constrained experiences may reduce the cognitive practice necessary for developing flexible reappraisal capacities (Dennis & Vander Wal, 2010). Given that cognitive flexibility develops through practice and environmental scaffolding (Diamond, 2013), educators who experienced such responses in childhood may exhibit more constrained cognitive repertoires, which could increase their reliance on rigid interpretive frames when responding to children’s negative emotions.

Building on these two distinct mediating patterns, the sequential mediation analysis revealed how subjective well-being and cognitive flexibility operate jointly. The study identified a significant sequential mediating pattern, in which childhood unsupportive experiences were associated with lower subjective well-being, which in turn was associated with reduced cognitive flexibility, and ultimately with higher levels of current unsupportive responses to children. This finding extends existing research by identifying a sequential affective–cognitive mechanism through which developmental experiences shape professional caregiving behavior. Consistent with Fredrickson’s (2001) broaden-and-build theory—which posits that positive affect expands cognitive repertoires—diminished well-being may constrain cognitive resources, which may in turn limit educators’ capacity to generate alternative interpretations when responding to children’s emotional expressions. From a neurocognitive perspective, Wang et al. (2017) experimentally demonstrated that positive emotions enhance cognitive flexibility while negative emotions impair it, offering empirical evidence that emotional valence directly modulates cognitive processing. While laboratory findings may not directly translate to the complex dynamics of classroom settings, they provide convergent evidence for the emotion–cognition link identified in the present study. It should be noted, however, that the cross-sectional design does not permit us to establish the temporal precedence of subjective well-being over cognitive flexibility. Alternative directional models—such as one in which higher cognitive flexibility fosters greater subjective well-being—remain theoretically plausible and were not tested in the present study. Future longitudinal designs are needed to adjudicate between competing directional accounts.

Although no prior study has tested the specific sequential pathway examined here, research with similar structural frameworks supports the plausibility of such mechanisms. Jang and Hong (2024) identified a sequential pathway from perceived maternal parenting to educator burnout through self-compassion and efficacy among Korean early childhood educators. Regarding affective outcomes, Peter et al. (2025) found that recalled parental emotion responses predicted adult depression through emotion beliefs and cognitive reappraisal in sequence. Most directly relevant, Girod et al. (2025) demonstrated that mothers’ recalled emotionally responsive parenting predicted maternal sensitivity through a serial pathway from negative emotional reactions to negative cognitive attributions about infant crying. Across these studies, the broadly consistent affective-to-cognitive directionality—spanning different populations, outcome variables, and caregiving contexts—strengthens the theoretical rationale for the sequential pathway identified in the present study.

The cultural context may offer one tentative, hypothesis-generating interpretation of how this sequential mechanism operates among Korean educators. It is possible that cultural norms of emotional restraint, characteristic of highly collectivistic contexts (Choi & Lee, 2025a; Hofstede et al., 2010), enable educators to maintain surface-level professional composure while not fully buffering the downstream cognitive consequences of diminished well-being (Choi & Lee, 2025b). This speculative account is consistent with the pattern of findings and may help illuminate two seemingly contradictory results: the non-significant direct pathway from well-being to unsupportive responses, and the significant indirect pathway through cognitive flexibility. However, because no cultural variables were directly measured in this study, this interpretation should be treated as a hypothesis to be examined rather than an explanatory conclusion. Future research that explicitly incorporates measures of collectivistic values, emotional display norms, and face-saving orientations would be needed to evaluate whether cultural context moderates the pathways identified here.

Alongside these interpretive considerations, it is important to contextualize the magnitude of the observed effects. The regression models in the present study explained between 17% and 25% of the variance in the outcome variables (*R*^2^ = 0.17–0.25). While statistically significant, these figures indicate that a substantial portion of variance in educators’ professional responses remains unaccounted for. This is expected given the inherent complexity of professional caregiving behavior, which is shaped by numerous additional factors, including real-time classroom demands, institutional support structures, collegial relationships, and individual personality characteristics, that were not captured in this study. The effect sizes should therefore be interpreted as modest but theoretically meaningful, positioning childhood experience and psychological resources as one contributory pathway among many. From a practical standpoint, however, even modest associations between early developmental experiences and professional behavior are noteworthy at the policy level: if attending to educators’ psychological histories and well-being yields even incremental improvements in the quality of teacher–child interactions, the cumulative developmental benefit to children across classrooms may be meaningful. From this perspective, well-being may shape educators’ professional responses not directly but by providing the affective foundation upon which cognitive flexibility operates.

## 6. Conclusions and Limitations

### 6.1. Limitations

This study has several limitations. First, all data were self-reported from a single source. As an initial screening for potential common method bias, Harman’s single-factor test was conducted, in which all items from the four measurement instruments were subjected to an exploratory factor analysis with a single unrotated factor extraction. The single factor accounted for only 18.42% of the total variance, well below the conventional 50% threshold. However, Harman’s test is widely recognized as an insensitive diagnostic tool that can fail to detect common method variance even when it is present (Podsakoff et al., 2003). Accordingly, common method bias remains a meaningful concern that this test cannot adequately address. Future research employing procedural remedies would provide more robust evidence; these include temporal separation of measurements, the use of independent raters, and multi-source designs. Two related self-report concerns also warrant mention: the CCNES measure of maternal responses relies on adult recall, and unmeasured dispositional factors, such as general affective dispositions and personality traits, may have influenced both the recall of maternal responses and current professional behaviors. That said, retrospective measures of childhood experiences retain strong predictive validity for adult psychological outcomes (Baldwin et al., 2019, 2024), and the sequential nature of the mediation pattern identified in the present study is difficult to attribute to any single unmeasured dispositional factor. Observational or longitudinal approaches would provide stronger evidence. Second, the cross-sectional design precludes causal conclusions. PROCESS (Model 6) tests mediation within correlational data and does not establish temporal precedence. Third, the sample was drawn exclusively from the South Korean ECE system, which operates under a centralized credentialing framework and a standardized national curriculum (Byun et al., 2022). These system-level features may shape how educators’ developmental histories relate to their professional practice. Replication in structurally different ECE systems would help clarify the generalizability of the proposed model.

### 6.2. Contributions

Despite these limitations, this study offers several important contributions. First, it empirically investigates the developmental and psychological mechanisms underlying early childhood educators’ unsupportive responses—an area that has received limited attention compared to positive interaction research. By introducing a sequential mediation framework, this study advances the understanding of how childhood experiences of maternal unsupportive responses influence current professional practice through an affective–cognitive pathway involving subjective well-being and cognitive flexibility.

Second, this study demonstrates that the influence of emotional well-being on professional responsiveness is not direct but is realized through the mediation of cognitive resources. This reconceptualization is particularly relevant in early childhood education, where sensitive responding depends not only on affective states but also on the cognitive capacity to interpret children’s emotions flexibly—a link supported by the integration of broaden-and-build theory (Fredrickson, 2001) with the emotional socialization framework (Eisenberg et al., 1998).

Third, from a policy and training perspective, the sequential mediation pathway suggests that interventions should be developmentally sequenced: first fostering emotional stability through institutional support and guided self-reflection on childhood emotional experiences, then building cognitive flexibility through perspective-taking and reappraisal training. Future intervention studies that experimentally target this sequential pathway would provide a direct test of the proposed causal mechanism.

## Figures and Tables

**Table 1:** Demographic characteristics of participants (*N* = 302).

Characteristics	Category	Frequency	Percentage
Age	20 s	62	20.6
	30 s	130	43.2
	40 s or older	109	36.2
Educational attainment	high-school graduates	16	5.3
	2–3-year college graduates	93	30.9
	4-year university graduates	146	48.5
	graduate school graduates	46	15.3
Teaching experience	less than 5 years	51	16.9
	5–9 years	77	25.5
	10–14 years	102	33.8
	15 years or more	72	23.8
Institution type	public kindergartens	97	32.1
	private kindergartens	63	20.9
	public childcare centers	82	27.2
	private childcare centers	37	12.3
	workplace/corporate childcare centers	23	7.6
Class assignments	3-year-old classes	94	31.1
	4-year-old classes	62	20.5
	5-year-old classes	75	24.8
	mixed-age classes	71	23.5
Total		302	100.0

**Table 2:** Descriptive statistics and correlations.

Variable	1	2	3	4	5	6	7
1. Age							
2. Education level	0.05						
3. Teaching experience	0.60 ***	0.13 *					
4. Maternal unsupportive responses in childhood	0.10	−0.10	0.01				
5. Subjective well-being	0.12 *	0.23 ***	0.14 *	−0.34 ***			
6. Cognitive flexibility	0.04	0.17 **	0.07	−0.27 ***	0.47 ***		
7. Unsupportive responses to young children	0.10	−0.09	−0.05	0.39 ***	−0.21 ***	−0.37 ***	
Min	23.00	1.00	1.00	1.27	1.00	2.55	1.20
Max	61.00	4.00	41.00	4.30	7.00	4.95	3.53
*M*	37.80	2.74	10.66	2.57	5.33	3.88	1.90
*SD*	8.37	0.78	6.15	0.58	1.06	0.47	0.45
Skewness	0.54	−0.19	1.07	0.47	−1.20	−0.14	0.92
Kurtosis	−0.42	−0.35	2.14	0.01	1.63	−0.08	0.72

*Note*. *N* = 302. * *p* < 0.05; ** *p* < 0.01; *** *p* < 0.001. 1: Age; 2: Education level; 3: Teaching experience; 4: Maternal unsupportive responses in childhood; 5: Subjective well-being; 6: Cognitive flexibility; and 7: Unsupportive responses to young children.

**Table 3:** Regression of maternal unsupportive responses on educator responses.

	*B*	β	*SE*	*t*	95% CI [LL, UL]	Tolerance	VIF
Age	0.01	0.17	0.00	2.51 *	[0.00, 0.02]	0.58	1.71
Education level	−0.02	−0.03	0.03	−0.56	[−0.08, 0.04]	0.97	1.03
Teaching experience	−0.01	−0.16	0.01	−2.37 *	[−0.02, 0.00]	0.58	1.72
Maternal unsupportive responses in childhood	0.29	0.37	0.04	6.93 ***	[0.21, 0.37]	0.98	1.02
	*F* = 15.47 ***, *R*^2^ = 0.17						

*Note*. *N* = 302. * *p* < 0.05; *** *p* < 0.001. *B* unstandardized regression coefficient; β, standardized regression coefficient; *SE*, standard error; *t*, *t*-test statistic; UL, upper level; CI, confidence interval; LL, lower level; *F*, *F*-statistic from ANOVA; *R*^2^, coefficient of determination; VIF, variance inflation factor.

**Table 4:** Sequential mediating effects.

Path			*B*	β	*SE*	*t*	95% CI [LL, UL]
SW	←	Age	0.01	0.10	0.01	1.44	[−0.01, 0.03]
		Education level	0.23	0.17	0.07	3.19 **	[0.09, 0.38]
		Teaching experience	0.01	0.07	0.01	1.02	[−0.01, 0.04]
		MUR	−0.61	−0.33	0.10	−6.20 ***	[−0.81, −0.42]
			*F* = 15.39 ***, *R*^2^ = 0.17				
CF	←	Age	0.00	−0.01	0.00	−0.19	[−0.01, 0.01]
		Education level	0.04	0.06	0.03	1.21	[−0.02, 0.10]
		Teaching experience	0.00	0.03	0.01	0.40	[−0.01, 0.01]
		MUR	−0.10	−0.13	0.04	−2.30 *	[−0.19, −0.02]
		SW	0.18	0.41	0.03	7.23 ***	[0.13, 0.23]
			*F* = 18.06 ***, *R*^2^ = 0.24				
UR	←	Age	0.01	0.18	0.00	2.69 ***	[0.00, 0.02]
		Education level	0.00	0.00	0.03	0.08	[−0.06, 0.06]
		Teaching experience	−0.01	−0.15	0.01	−2.26 *	[−0.02, 0.00]
		MUR	0.24	0.30	0.04	5.53 ***	[0.15, 0.32]
		SW	0.01	0.03	0.03	0.52	[−0.04, 0.06]
		CF	−0.28	−0.30	0.06	−5.14 ***	[−0.39, −0.17]
			*F* = 16.10 ***, *R*^2^ = 0.25				

*Note*. *N* = 302. * *p* < 0.05; ** *p* < 0.01; *** *p* < 0.001. *B* unstandardized regression coefficient; β, standardized regression coefficient; *SE*, standard error; *t*, *t*-test statistic; CI, confidence interval; UL, upper level; LL, lower level; *F*, *F*-statistic from ANOVA; *R*^2^, coefficient of determination; “←” indicates the criterion variable (left) regressed on the predictors (right). MUR, maternal unsupportive responses in childhood; SW, subjective well-being; CF, cognitive flexibility; UR, unsupportive responses to young children.

**Table 5:** Specific indirect effects of maternal unsupportive responses in childhood on unsupportive responses to young children.

Path	Effect	Boot *SE*	β	95% CI [LL, UL]
Total indirect	0.05	0.03	0.07	[0.02, 0.12]
UR ← SW ← MUR	−0.01	0.02	−0.01	[−0.06, 0.03]
UR ← CF ← MUR	0.03	0.02	0.04	[0.00, 0.07]
UR ← CF ← SW ← MUR	0.03	0.01	0.04	[0.02, 0.07]

*Note*. *N* = 302. Effect, unstandardized indirect effect; Boot *SE*, bootstrap standard error; β, completely standardized indirect effect; CI, bootstrap confidence interval; LL, lower level; UL, upper level. MUR, maternal unsupportive responses in childhood; SW, subjective well-being; CF, cognitive flexibility; UR, unsupportive responses to young children.

## Data Availability

The data presented in this study are available upon request from the corresponding author due to privacy concerns.

## Abbreviations

The following abbreviations are used in this manuscript:

MUR	Maternal unsupportive responses in childhood
SW	Subjective well-being
CF	Cognitive flexibility
UR	Unsupportive responses to young children
CCNES	Coping with children’s negative emotion scale
CCNES-T	Coping with children’s negative emotion scale–teacher
SHS	Subjective happiness scale
